# Supplementary material for: Schistosomiasis amongst adolescent boys in non-lakeshore southern Malawi: Investigating local risk-factors within a nested community-based cross-sectional survey
Source: PLoS Negl Trop Dis. 2025 Dec 1;19(12):e0013745. doi: 10.1371/journal.pntd.0013745 (PMC12668493; doi:10.1371/journal.pntd.0013745)
Supplement: S1 File — (DOCX) [file pntd.0013745.s001.docx]

S1 File: Questionnaire structure and translation

| **Do you think you are currently infected with either schistosomiasis or bilharzia or a worm infestation?** | Mukuganiza kuti muli ndi tizilombo toyambitsa likodzo? |
| --- | --- |
| **Do you currently have any blood in your urine or red urine?** | Pakanali pano,mukamakodza mkodzo wanu ukumatuluka wa magazi? |
| **Do you currently have any blood in your stool?** | Kodi pakanali pano,mukamachita chimbudzi,mukumapezeka magazi ena aliwonse? |
| **Have you been swimming in any water bodies in the past week?** | Kodi pa masabata apitawa,munasambako ku mtsinje,ku Nyanja kapena kumadambo? |
| **Did you wash any dishes or clothes in any water bodies over the last week?** | Kodi sabata lapitali,munatsukako mbale kapena kuchapa zovala ku malo omwe ndatchulawa? |
| **Did you wash any part of your body in any water bodies over the last week?** | Kodi sabata lapitali,munapitako Kumalo ndatchulawa kukasamba? |
| **Did you play in any water bodies over the last week?** | Munasewerako malo ndatchulawa sabata lathali? |
| **Did you go fishing in any water bodies over the last week?** | Masabata apitawa munakawedzako ku mmalo ndatchulawa? |
| **How many full days of school have you attended in the past 7 days?** | Fuso langa lomaliza,sabata lapitali munapitako kangati ku sukulu mosadukiza? |
