# Supplementary figures and images for: Schistosomiasis amongst adolescent boys in non-lakeshore southern Malawi: Investigating local risk-factors within a nested community-based cross-sectional survey
Source: PLoS Negl Trop Dis. 2025 Dec 1;19(12):e0013745. doi: 10.1371/journal.pntd.0013745 (PMC12668493; doi:10.1371/journal.pntd.0013745)

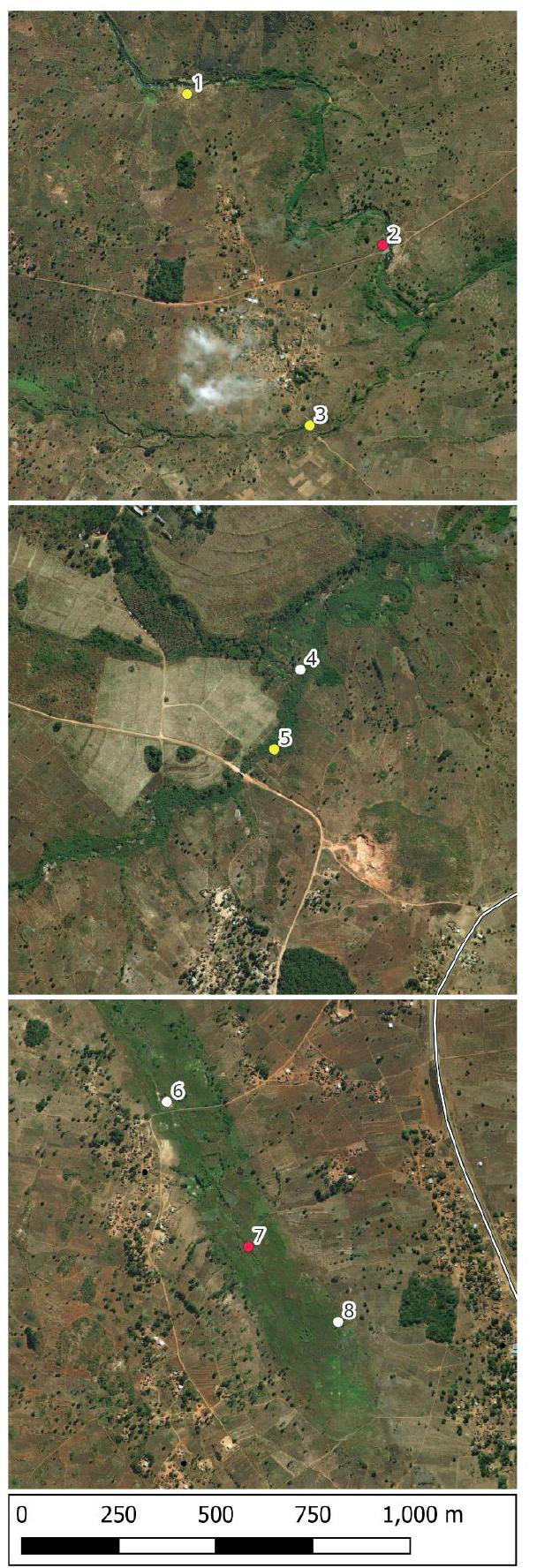

Supplement: S1 Fig — Contains information from World Imagery (Esri, DigitalGlobe, GeoEye, i-cubed, USDA FSA, USGS, AEX, Getmapping, Aerogrid, IGN, IGP, swisstopo, and the GIS User Community). Map image is the intellectual property of Esri and is used herein under license. Copyright 2025 Esri and its licensors. All rights reserved. Map base layer; [https://www.arcgis.com/home/item.html?id=10df2279f9684e4a9f6a7f08febac2a9]. Terms of use; [https://doc.arcgis.com/en/arcgis-online/reference/static-maps.htm], [https://support.esri.com/en-us/knowledge-base/what-is-the-correct-way-to-cite-an-arcgis-online-basema-000012040#:~:text=When%20an%20ArcGIS%20Online%20basemap,task%2C%20or%20application%20being%20used], [https://content.esri.com/arcgisonline/docs/tou_summary.pdf]. (TIF) [file pntd.0013745.s002.tif]

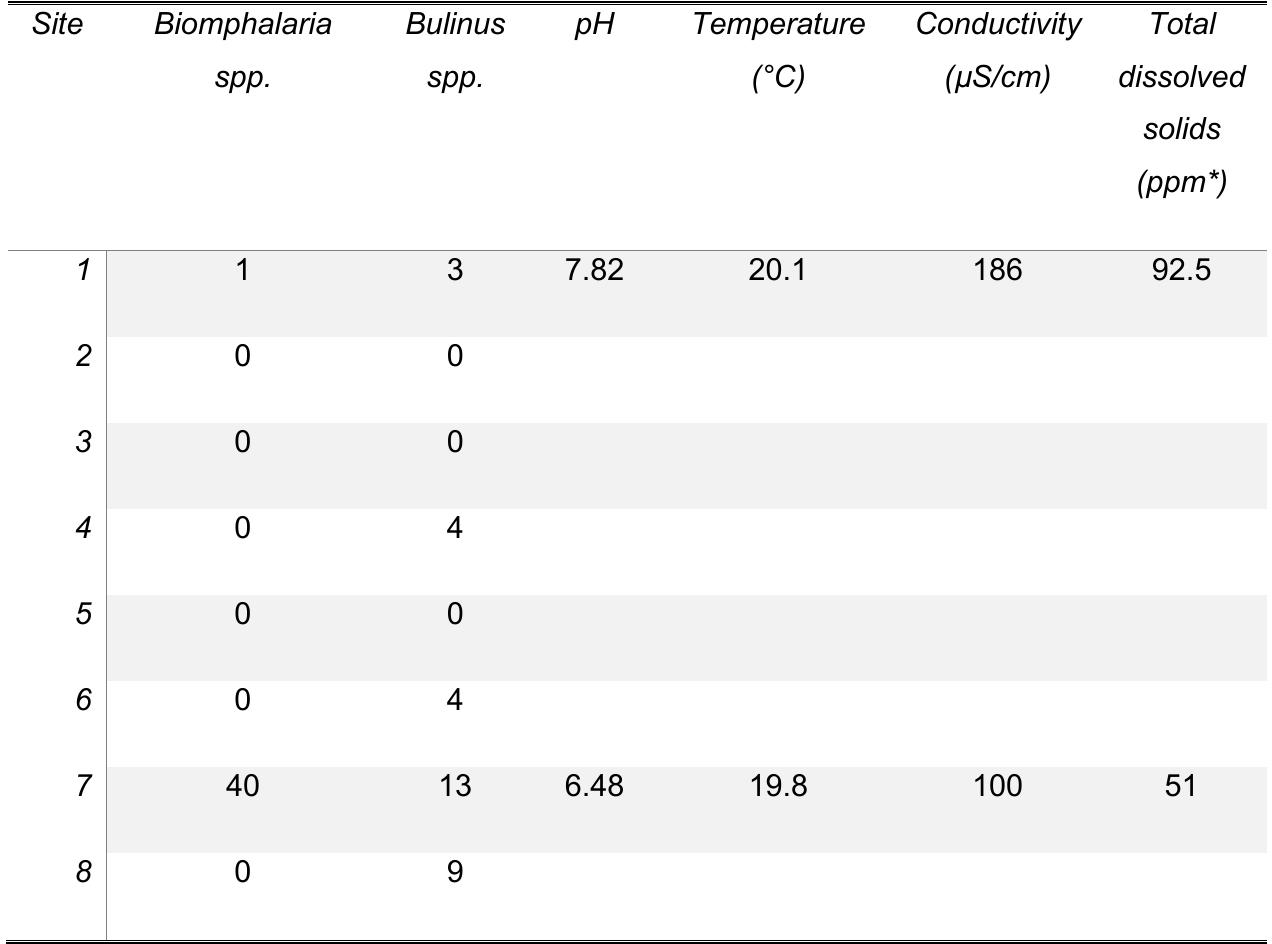

Supplement: S2 Fig — (*) Parts per million. (TIF) [file pntd.0013745.s003.tif]
