# Supplementary material for: Schistosomiasis amongst adolescent boys in non-lakeshore southern Malawi: Investigating local risk-factors within a nested community-based cross-sectional survey
Source: PLoS Negl Trop Dis. 2025 Dec 1;19(12):e0013745. doi: 10.1371/journal.pntd.0013745 (PMC12668493; doi:10.1371/journal.pntd.0013745)
Supplement: Attachment — Submitted filename: Response to reviewers_sept2025 SWM.docx [file pntd.0013745.s006.docx]

Friday 10^th^ October 2025

**Response to reviewers - PNTD-D-25-00833**

Dear Editors,

We thank both reviewers for their comments and have revised the manuscript accordingly. Please find our specific responses to each point below:

**Response to reviewer 1 (‘Editorial and Data Presentation Modifications’ section):**

*1. Observed weaknesses/Limitations of the study- limited age/sex scope: The study focuses solely on male participants aged 10–15, excluding girls and other age groups who may also bear significant burden.*

We have further justified the reasoning for focusing on 10-15 year old boys in section 4.3:

“**Participants 10 to 15 years old were chosen as they generally exhibit the heaviest infections and highest prevalence of the community [13], with boys specifically as microscopic haematuria in principle should demonstrate the best positive predictive value in this group** **as haematuria of other aetiologies is less common (such as menstruation or neoplasm).**”

*2. Low coverage of stool and urine sampling: Only 243 out of 306 participants provided urine samples, and a slightly smaller number completed the risk factor questionnaire, potentially introducing bias or missingness in the risk analysis.*

We have now incorporated this reflection on selection bias into the discussion section:

“**There are a number of sources of potential selection bias in our study. Given Kato-Katz and urine sampling only occurred for a limited time within a larger survey, study clusters were unevenly sampled. Additionally, only 79% of participants were revisited to obtain urine, due to resource constraints**.”

*3. No serological or antigen-based diagnostics (e.g., POC-CCA or UCP-LF CAA) were included, which limits sensitivity for light infections and hampers comparability with other community-based mapping studies.*

We note the reviewers point, but highlight that we have already mentioned in the final paragraph of the discussion section that any further studies in this area should include CCA:

**“We recommend a low cost, community wide survey using urine reagent strips and CCA is promptly conducted, integrated with a questionnaire regarding MDA coverage”**

We have also mentioned in our discussion section, whilst discussing limitations of our parasitological methods that CCA may have been a better choice of screening tool than KK:

**“Given these limitations CCA may have been a more appropriate** diagnostic **method in this population**

4. *Cross-sectional design limits inference on causality or seasonality, especially as the survey was conducted over just two months (July–August).*

We have now incorporated both these limitations within the discussion section:

“**Importantly, this study is unable to account for seasonality of transmission and by being cross sectional it limits any inference of causality**.”

5. *Limited discussion of potential reasons for high S. haematobium despite MDA, such as water contact behaviour patterns, sanitation access, or treatment adherence.*

We have expanded the discussion section to include these potential reasons for high prevalence:

“**Human factors such as the prevalence of recent high risk water contact or inadequate WASH intervention may also be leading to high re-infection rates.**”

6. *Broaden risk-factor analysis: Include multivariable regression results (even if not significant) to show which behaviors or exposures were most associated with infection.*

We thank the reviewer for their suggestion. The analysis presented was intended to focus specifically on reported water contact behaviours and school attendance as predictive factors for infection. While a secondary analysis that incorporates a broader range socio-demographic characteristics (e.g. SES, parental education) or other factors (e.g. recent treatment with PZQ, distance to water body) could provide additional insights, this was beyond the scope of our targeted investigation of behavioural risk factors.

7. *Expand discussion on MDA limitations: The manuscript should further explore why MDA has not reduced heavy infections, discussing factors like coverage accuracy, treatment efficacy, reinfection rates, or drug delivery strategy.*

We have included in our discussion statement two statements when examining reasons for high prevalence:

**“Human factors such as the prevalence of recent high risk water contact or inadequate WASH intervention may also be leading to high re-infection rates. Though praziquantel resistance is not reported, it must also be considered whether treatment efficacy is lower than anticipated due to host, parasite, or MDA regimen factors.”**

8. *Clarify malacological survey: More detail is needed on snail infection status, cercarial shedding, or snail density trends—critical to linking human and environmental transmission.*

We have now revised the discussion section to include the need for this evidence, as part of future research:

“**A more thorough malacological survey is necessary to establish snail density trends and infection prevalence in this area and evaluate the need for snail control programmes.**”

9. *Consider including females in future work: Gendered differences in water contact and school attendance could influence transmission risk. There are no communities with only males only exclusion of the other gender in any study is already bias driven.*

We thank the reviewer for this comment, which we have taken as a (appropriate) suggestion for future work, and highlight that in our conclusion have stated that any further work should be community wide rather than a focus only on a specific demographic group.

10. *Strengthen conclusions with policy relevance: Offer concrete recommendations, such as inclusion of non-lakeshore areas in control programs, WASH interventions, or entomological surveillance expansion.*

We have revised our concluding paragraph to include stronger recommendations for policy:

“**Both a more thorough water exposure and malacological survey are required to inform non-MDA interventions. Our study highlights the need for non-lake shore communities previously regarded as ‘low risk’ to be included in control programmes and national surveys.** We re-emphasise the importance of investment in epidemiological monitoring alongside schistosomiasis MDA programmes, and that this monitoring can be effectively conducted with low-cost screening methods and with minimal technical staff.”

**Response to reviewer 2:**

**Abstract:**

*Line 47: Please change “this” to “these”*

We have left this unchanged, as we believe it is clearer as currently written.

**Introduction:**

*Line 80: please write “…. chemotherapy by annual mass drug administration (MDA)….” Instead of “… chemotherapy by annual MDA (mass drug administration)…”*

*Line 80: write “… if schistosomiasis prevalence is shown to be >50% amongst school age children (SAC)…” instead of “…. if schistosomiasis prevalence is shown to be >50% amongst SAC (school age children).*

*Lines 94, 96 and 103: “Bulinus”, “Biomphalaria” and “Schistosoma” should be italicized.*

These three points have been revised accordingly.

*Lines 119-120: “The primary objective … amongst 10–15-year-old boys in Namwera … Could the authors please explain why this specific age and sex group was chosen for this objective?*

This comment has been addressed by revisions to section 4.3:

“This study’s sample size was determined based on the expected prevalence of microscopic haematuria. Participants **10–15 years old were chosen as they generally exhibit the heaviest infections and highest prevalence of the community [13], with boys specifically as microscopic haematuria in principle should demonstrate the best positive predictive value in this group** **as haematuria of other aetiologies is less common (such as menstruation or neoplasm).” .**”

*Lines 124-127: “To our knowledge … the presence of intermediate host snail species of schistosomiasis in the area”. Why limit the study to the presence of snails only? It would have been valuable to assess their actual involvement in transmission by conducting cercarial shedding tests.*

We agree with the reviewer’s comment, however, cercarial shedding was not able to be performed in this study due to limitations in both expertise and resources. Though we do not feel it is necessary to specifically state this in the paper, we have made a recommendation in the discussion section that it is done as part of further studies:

“**A more thorough malacological survey is necessary to establish snail density trends and infection prevalence in this area, and evaluate the need for snail control programmes.**”

**Methods:**

*Lines 144-145: “Entire communities … between 1650 to 4000 individuals”. I suggest adding a brief description of how the communities were delineated into these clusters, or providing a citation to a previous publication where this process is described in detail. Additionally, I recommend rephrasing “Entire communities … between 1650 to 4000 individuals” as “Entire communities … between 1650 and 4000 individuals”.*

We have revised the manuscript to better describe how the study clusters were delineated (section 4.1):

“**Following a site-wide census**, communities **(villages)** were delineated into 40 study clusters of between 1650 **and** 4000 individuals. **Where possible the study cluster preserved the Health Surveillance Assistant catchment area, with one or more villages combined to form each cluster, and no villages subdivided.**”

*Line 173: “... a sample size of 203 participants …”. Could the authors please provide the formula used to calculate this sample size, along with the assumptions or parameters?*

The necessary parameters for this calculation are provided in the text, however, we have now provided a reference to the software and function used to calculate this (section 4.3):

“The prevalence of S. haematobium was assumed to be 24%, based on a recent nearby school based parasitological survey [11], and it was assumed a prevalence of S. haematobium of 24% would translate to an identical prevalence of microhaematuria [25]. Using a confidence limit (absolute precision) of 5.5%, a design effect of 1, and a confidence level of 95%, a sample size of 203 participants was required. **This was calculated using OpenEpi (Sample Size for Frequency in a Population) [26].**”

*Lines 180-181: “This list randomly …, and a maximum number set …”. Please clarify how this number was determined.*

We have now clarified that this number was determined by randomly ranking all eligible samples and setting the number to be assessed based on lab capacity on each day (section 4.4):

“At the start of each day where Kato-Katz was performed, **a sampling list of eligible stool samples collected the previous day was generated** in Stata 17 (College Station, TX: StataCorp LLC) **and samples randomly ranked within study cluster. From this list, a maximum number was set for how many samples could be analysed from each study cluster,** according to laboratory capacity.”

*Line 186: “Terminal urine samples were collected approximately 2 weeks after stool”. Please provide the reason for the 2-week interval between stool and urine sample collection.*

We have now stated that this was due to delays in obtaining equipment:

“Terminal urine samples were collected approximately 2 weeks after stool **due to delays in obtaining equipment.**”

*Lines 201 and 338: Please place appendix 1 before appendix 2 to ensure proper sequencing.*

This has been corrected.

*Line 213: “… using convenience sampling …”. Could the authors please provide more details about how these communities were selected?*

We have now clarified that this was based on where the parent study was working at said time (section 4.6):

“Communities were then selected using convenience sampling **based on whether the parent study was working in said community at that time**.”

*Line 218: “Snails were collected for one hour at each site …”. Please specify how many people carried out the snail collection at each site?*

We have clarified that it was just one person (section 4.6):

“Snails were collected **by one person** for one hour at each site using a sieve attached to a rod as well as handpicking from vegetation using gloves.”

*Line 227: “Age was deemed to be an a priori confounder …”. Please explain why age was considered a confounder. Also, delete "an" so the sentence reads "… a priori confounder…."*

We have now stated in section 4.3 that infection prevalence and intensity (and water exposure) peaks at 10-15 years old. We have also corrected the sentence as suggested. Additionally, we have attempted to justify why age confounds the relationship between infection and haematuria (line 265) elsewhere by mentioning haematuria is less common in younger people.

**“Age was deemed to be a confounder a priori”**

**“Participants 10 to –15 -years -old were chosen as they generally exhibit the heaviest infections and highest prevalence of the community, in part due to frequent water exposure [13],”**

“with boys specifically as microscopic haematuria in principle should demonstrate the best positive predictive value in this group as haematuria of other aetiologies is less common (such as menstruation or neoplasm)”

*Lines 242-243: “… terms of sensitivity, specificity, negative predictive value (NPV) and positive predictive value (PPV) …” I suggest adding the calculation formulas for each of these terms (sensitivity, specificity, NPV, and PPV) to help readers better understand the diagnostic performance assessment*

This has been done as suggested (section 4.7):

“The performance of urine reagent strip parameters was assessed against urine filtration in terms of sensitivity **(percentage of infected participants with a positive test result**), specificity (**percentage of uninfected participants with a negative test result**), negative predictive value (**NPV= number of uninfected participants with a negative test/ (number of uninfected participants with a negative test + number of infected with a negative test**) and positive predictive value (**PPV= number of infected participants with a positive test / (number of infected participants with a positive test+ number of uninfected participants with a positive test**) for detecting S. haematobium infection.”

**Results:**

*Lines 260-261: “… 16 (5.1%) were readable on three slides and 272 (86.7 %) were readable on all four slides”. In Methods section (Line 182), it is mentioned that two slides were prepared per stool sample. Could the authors please clarify how many slides were actually prepared per sample for this analysis? There seems to be an inconsistency that needs to be addressed.*

We have now clarified in parasitological methods (section 4.4) that pairs of slides were read first at 30 to 60 minutes and re-read 18 to 24 hours later, resulting in four slide readings per sample in total.

“In-line with parent study protocols and WHO recommendations, two slides were prepared and read from each sample at 30 to 60 minutes after preparation, both slides were read a second time after 18-24 hours to better visualise Schistosoma spp eggs [27, 28].”

*Line 266: “S. mansoni” should be italicized.*

*Line 272: For the first appearance of species names, please write them in full (i.e., Hymenolepis diminuta instead of H. diminuta and Ascaris lumbricoides instead of A. lumbricoides).*

These two points have been revised.

*Line 273: “Five participants (1.6%) had S. haematobium infections on Kato-Katz…”. Given that the Kato-Katz technique is typically used for diagnosing S. mansoni, I suggest providing an explanation for this finding in the Discussion section.*

This has been addressed in paragraph two of the discussion section, with a reference:

“**The five cases of S. haematobium demonstrated on Kato-Katz likely represent ectopic infection due to ‘spill-over’ of heavy infection or heterospecific mating. All Schistosoma spp. eggs in urine were assumed to be S. haematobium, it is possible ectopic S. mansoni infections were present [32].**”

*Lines 297-300: “Using a random effects logistic regression model adjusted…”. For clarity and to help readers better follow the results, please consider presenting the regression results in a table.*

We recognise the reviewers concern, but feel that an extra table might be too many, and that the paragraph is sufficiently easy to understand without this. We also feel this information is less important than sensitivity and specificity, and therefore do not wish to emphasise they results.

*Lines 313-317: ““Blood in stool” demonstrated …. detecting S. mansoni infection”. To improve readability, I suggest presenting these results in the same format as the previous paragraph (lines 306-312), including confidence intervals and ideally summarizing them in a table*

This section has been revised as suggested for consistency and to include the confidence intervals, however, as with the previous point we feel that these results remains best presented in the text rather than as a table.

“Blood in stool” demonstrated a sensitivity of 6.7% **(95% CI: 0.2-31.9)**, a specificity of 83.9% **(95% CI: 77.9-88.8)**, PPV of 3.1% **(95% CI: 0.1-16.2)**, NPV of 92.0% **(95% CI: 86.9-95.6)** and a spearman rank p-value of 0.34 compared to Kato-Katz in detecting S. mansoni infection. “Bilharzia” demonstrated a sensitivity of 53.9% **(95% CI: 25.1-80.8)**, specificity of 48.1% **(95% CI: 40.7-55.6)**, PPV of 6.8% **(95% CI: 2.8-13.5)**, NPV of 93.7% **(95% CI: 86.8-97.6)** and a spearman rank p-value of 0.93 compared to Kato-Katz in detecting S. mansoni infection.

*Line 321: “An LRT conducted using our….”. Please write “LRT” in full.*

This has been revised.

*Lines 323-324: “Additionally, …. between age in years and intensity of S. haematobium infection (p=0.29)”. This does not appear in any table.*

We have removed this erroneous statement.

*Line 338: “Water readings were taken at the two Biomphalaria spp. sites”. Why were water measurements taken only at the two Biomphalaria spp. sites and not at all sampling sites? Also, please do not italicize “spp.”*

We have revised section 5.6 to clarify the reason for this:

“Water readings were only taken at the two *Biomphalaria* spp*.* sites several weeks after snail collection **due to limited access to a digital water tester** (supplementary figure 2).”

References:

*Line 524: citation 23. Please add the authors’ names*

The authorship given for this article is correct, which was published using a (single) group authorship:

*DeWorm3 Trials Team. Baseline patterns of infection in regions of Benin, Malawi and India seeking to interrupt transmission of soil transmitted helminths (STH) in the DeWorm3 trial. PLoS Negl Trop Dis. 2020 Nov 2;14(11):e0008771. doi: 10.1371/journal.pntd.0008771.*

Tables:

*Table 1: I suggest removing the variable "Sex" as it is already known that the study focused exclusively on boys*

*Table 2: In column 3 (Number of participants with S. haematobium infection), please include the corresponding percentages in parentheses.*

These have both been revised.

Yours sincerely,

Dr. Oscar Herrera
