# Supplementary material for: Schistosomiasis amongst adolescent boys in non-lakeshore southern Malawi: Investigating local risk-factors within a nested community-based cross-sectional survey
Source: PLoS Negl Trop Dis. 2025 Dec 1;19(12):e0013745. doi: 10.1371/journal.pntd.0013745 (PMC12668493; doi:10.1371/journal.pntd.0013745)
Supplement: Attachment — Submitted filename: Reviewer Comments.pdf [file pntd.0013745.s004.pdf]

Reviewer Comments

PLOS Neglected Tropical Diseases

Manuscript Number: PNTD-D-25-00833

Manuscript Title: Schistosomiasis amongst adolescent boys in non-lakeshore southern Malawi: Investigating local risk-factors within a nested community-based cross-sectional survey

Authors: Oscar Herrera[1], Stefan Witek-McManus[1], James Simwanza[2], Lyson Samikwa[3], Stella Kepha[4], Rejoice Msiska[2], Sean Galagan[5], Elliott Rogers[1], Peter Makaula[6], J. Russell Stothard[7], Judd Walson[5], Lazarus Juziwelo[8†], Rachel Pullan[1], Khumbo Kalua[2,3], Robin Bailey[9]

Overview

I congratulate the authors on a clear and well-written paper that I think will be very interesting to the field. I have some minor suggestions for things that struck me as a reader that the authors could easily address.

ABSTRACT

Line 47: Please change “this” to “these”

INTRODUCTION

Line 80: please write “…. chemotherapy by annual mass drug administration (MDA)….” Instead of “… chemotherapy by annual MDA (mass drug administration)…”

Line 80: write "… if schistosomiasis prevalence is shown to be >50% amongst school age children (SAC)…" instead of "…. if schistosomiasis prevalence is shown to be >50% amongst SAC (school age children).

Lines 94, 96 and 103: "Bulinus", "Biomphalaria" and "Schistosoma" should be italicized.

Lines 119-120: "The primary objective … amongst 10–15-year-old boys in Namwera … Could the authors please explain why this specific age and sex group was chosen for this objective?

Lines 124-127: "To our knowledge … the presence of intermediate host snail species of schistosomiasis in the area". Why limit the study to the presence of snails only? It would have been valuable to assess their actual involvement in transmission by conducting cercarial shedding tests.

METHODS

Lines 144-145: "Entire communities … between 1650 to 4000 individuals". I suggest adding a brief description of how the communities were delineated into these clusters, or providing a citation to a previous publication where this process is described in detail. Additionally, I recommend rephrasing "Entire communities … between 1650 to 4000 individuals" as "Entire communities … between 1650 and 4000 individuals".

Line 173: "... a sample size of 203 participants …". Could the authors please provide the formula used to calculate this sample size, along with the assumptions or parameters?

Lines 180-181: "This list randomly …, and a maximum number set …". Please clarify how this number was determined.

Line 186: "Terminal urine samples were collected approximately 2 weeks after stool". Please provide the reason for the 2-week interval between stool and urine sample collection.

Lines 201 and 338: Please place appendix 1 before appendix 2 to ensure proper sequencing.

Line 213: "… using convenience sampling …". Could the authors please provide more details about how these communities were selected?

Line 218: "Snails were collected for one hour at each site …". Please specify how many people carried out the snail collection at each site?

Line 227: "Age was deemed to be an a priori confounder …". Please explain why age was considered a confounder. Also, delete "an" so the sentence reads "… a priori confounder… ."

Lines 242-243: "… terms of sensitivity, specificity, negative predictive value (NPV) and positive predictive value (PPV) …" I suggest adding the calculation formulas for each of these terms (sensitivity, specificity, NPV, and PPV) to help readers better understand the diagnostic performance assessment.

RESULTS

Lines 260-261: "… 16 (5.1%) were readable on three slides and 272 (86.7 %) were readable on all four slides". In Methods section (Line 182), it is mentioned that two slides were prepared per stool sample. Could the authors please clarify how many slides were actually prepared per sample for this analysis? There seems to be an inconsistency that needs to be addressed.

Line 266: "S. mansoni" should be italicized.

Line 272: For the first appearance of species names, please write them in full (i.e., *Hymenolepis diminuta* instead of *H. diminuta* and *Ascaris lumbricoides* instead of *A. lumbricoides*).

Line 273: "Five participants (1.6%) had *S. haematobium* infections on Kato-Katz…". Given that the Kato-Katz technique is typically used for diagnosing *S. mansoni*, I suggest providing an explanation for this finding in the Discussion section.

Lines 297-300: "Using a random effects logistic regression model adjusted…". For clarity and to help readers better follow the results, please consider presenting the regression results in a table.

Lines 313-317: ““Blood in stool” demonstrated …. detecting *S. mansoni* infection”. To improve readability, I suggest presenting these results in the same format as the previous paragraph (lines 306-312), including confidence intervals and ideally summarizing them in a table.

Line 321: “An LRT conducted using our….”. Please write “LRT” in full.

Lines 323-324: “Additionally, …. between age in years and intensity of *S. haematobium* infection (p=0.29)”. This does not appear in any table.

Line 338: “Water readings were taken at the two *Biomphalaria spp.* sites”. Why were water measurements taken only at the two *Biomphalaria* spp. sites and not at all sampling sites? Also, please do not italicize “spp.”

REFERENCES

Line 524: citation 23. Please add the authors’ names.

Tables

Table 1: I suggest removing the variable "Sex" as it is already known that the study focused exclusively on boys.

Table 2: In column 3 (Number of participants with *S. haematobium* infection), please include the corresponding percentages in parentheses.
